# Impact of Prior Statin Use on Reperfusion Rate and Stroke Outcomes in Patients Receiving Endovascular Treatment

**DOI:** 10.3390/jcm10215147

**Published:** 2021-11-02

**Authors:** Sang-Hwa Lee, Min Uk Jang, Yerim Kim, So Young Park, Chulho Kim, Yeo Jin Kim, Jong-Hee Sohn

**Affiliations:** 1 Department of Neurology, Chuncheon Sacred Heart Hospital, Hallym University College of Medicine, Chuncheon 24253, Korea; bleulsh@naver.com (S.-H.L.); gumdol52@naver.com (C.K.); yjhelena@hanmail.net (Y.J.K.); 2 Institute of New Frontier Research, Hallym University, Chuncheon 24252, Korea; 3 Department of Neurology, Dongtan Sacred Heart Hospital, Hallym University College of Medicine, Hwaseong 18450, Korea; mujang@gmail.com; 4 Department of Neurology, Kangdong Sacred Heart Hospital, Hallym University College of Medicine, Seoul 05355, Korea; brainyrk@gmail.com; 5 Department of Endocrinology and Metabolism, Kyung Hee University Hospital, Seoul 02447, Korea; malcoy@hanmail.net

**Keywords:** prior statin, endovascular treatment, reperfusion rate, early neurologic deterioration, stroke outcome

## Abstract

Background: We evaluated the impact of prior statin use on successful reperfusion and stroke outcomes after endovascular treatment (EVT). Method: Using consecutive multicenter databases, we enrolled acute ischemic stroke patients receiving EVT between 2015 and 2021. Patients were classified into prior statin users and no prior statin users after a review of premorbid medications. The primary outcome measure was successful reperfusion defined as modified TICI grade 2b or 3 after EVT. Secondary outcome measures were early neurologic deterioration (END) and a 3-month modified Rankin Scale (mRS) score of 0 to 2. Results: Among 385 patients receiving EVT, 74 (19.2%) were prior statin users, who had a significantly higher successful reperfusion rate compared with no prior statin users (94.6% versus 78.8%, *p* = 0.002). Successful reperfusion and END occurrence were improved according to statin intensity with a dose–response relationship. In multivariate analysis, prior statin was associated with successful reperfusion after EVT (adjusted odds ratio (95% confidence interval) 5.31 (1.67–16.86)). In addition, prior statin was associated with a lower occurrence of END and good functional status. Conclusion: Our study showed that prior statin use before ischemic stroke might improve successful reperfusion and stroke outcomes after EVT.

## 1. Introduction

Endovascular treatment (EVT) has an important role in improving stroke outcomes in patients with large vessel occlusion [1]. However, major clinical trials reported that 67% of patients undergoing EVT had poor outcomes despite successful reperfusion [2]. Reasons for this might be that reperfusion injury, re-occlusion, and no-reflow phenomenon are responsible for the poor outcomes [3]. To overcome this, several studies investigated which indicators influenced a good or poor prognosis in patients who underwent EVT [4,5].

Statin, an HMG Co-A reductase inhibitor, is used widely for the primary and secondary prevention of stroke [6]. Statins have pleiotropic effects, including anti-inflammatory, anti-thrombotic, and antioxidative effects, as well as cholesterol-lowering effects [7,8,9]. Therefore, in the acute stage of ischemic stroke, statins were expected to enhance thrombolytic effects, increase cerebral blood flow, and have neuroprotective effects that reduced reperfusion injury and prevented re-occlusion by suppressing MMP-9 [10,11,12,13]. Because oxidative stress and inflammatory cascades are widespread at the onset of acute stroke, we assumed that the use of statins would induce a good prognosis in patients receiving EVT. Most studies of early statin administration have focused on patients receiving IV thrombolysis (IVT), and studies in patients receiving EVT are lacking [14,15,16,17,18]. In addition, previous studies on EVT focused on the early administration of statins after EVT [19], whereas no studies have evaluated the effect of prior use of statins before a stroke on outcomes after EVT.

In the current real-world practice of statin use, which is continuously increasing [20,21], we aimed to investigate the effect of prior use of statins on reperfusion and outcomes in patients receiving EVT.

## 2. Methods

### 2.1. Study Population

We consecutively registered all acute ischemic stroke patients between March 2015 and March 2021 in three university-affiliated institutions (Chuncheon Sacred Heart Hospital, Dongtan Sacred Heart Hospital, and Kangdong Sacred Heart Hospital). We identified acute ischemic stroke patients treated with EVT. All the enrolled patients received acute stroke management according to institutional protocols based on recent guidelines. In this study, we excluded the following patients: (1) patients without followed-up brain computed tomography (CT) or magnetic resonance imaging (MRI) within 24 h of stroke onset; (2) patients with unavailable data regarding statin use; (3) patients with Alberta Stroke Program Early CT score (ASPECTS) <6; and (4) patients with a pre-stroke modified Rankin Scale (mRS) ≥ 2.

### 2.2. Data Collection and Definition of Parameters

We obtained the following data directly from the registry database: (1) demographics, including age and sex; (2) stroke risk factors, medical history, prior stroke, hypertension, diabetes mellitus (DM), hyperlipidemia, atrial fibrillation, current smoking status, pre-stroke status, and prior use of statin and antithrombotic drugs; (3) stroke characteristics, acute stroke treatment, initial NIHSS score, ischemic stroke mechanism according to the Trial of Org 10,172 in Acute Stroke Treatment (TOAST) classification with some modifications [22], tissue plasminogen activator (tPA) dose, and reperfusion therapy (IVT and EVT); and (4) laboratory data including hemoglobin, white blood cell count, platelet count, serum creatinine, initial random glucose, fasting low-density lipoprotein, prothrombin time, glycated hemoglobin (HbA1c), and systolic blood pressure.

All the premorbid medications of stroke patients were confirmed by the Department of Pharmacy of each institution. The premorbid medications were retrieved by nurses and physicians during hospitalization. We reviewed electronic medical records to ascertain the dose and type of statin used before stroke onset. Because statins are used in several forms and doses, we categorized these into low-, moderate-, and high-intensity statins (Supplementary Table S1) [23].

The primary outcome measure was successful reperfusion defined by modified Thrombolysis In Cerebral Infarction (mTICI) grade 2b to 3 [24]. Vascular neurologists in each institution made decisions on whether to perform reperfusion therapy. Regarding EVT procedures, the choice of devices and intervention strategies were at the discretion of interventionists in each institution. Secondary outcome measures were the occurrence of early neurologic deterioration (END) after EVT and good functional outcome at 3 months (mRS 0 to 2). END was defined as an increment of at least 1 point in motor power or a total NIHSS score deterioration of ≥2 points within 7 days of admission compared with the initial NIHSS score. The etiologies of END were classified into stroke recurrence (END-rec), stroke progression (END-prog), symptomatic hemorrhagic transformation (END-sHT), and others. END-rec was defined as any END with discrete new lesions on the diffusion-weighted image (DWI). END-prog was defined as END caused by the progression of initial ischemic lesion, which was the enlargement of infarct size or significant perilesional edema on follow-up images. END-sHT was defined as the presence of hemorrhagic transformation that explained END on follow-up images [25]. The infarct volume confirmed by DWI was calculated using ABC/2 method [26]. The inter-rater reliability for the evaluation of infarct volume was good (ICC 0.81, *p* < 0.001).

### 2.3. Statistical Analysis

Summary statistics are presented as the number of subjects (percentage) for categorical variables and as the mean ± SD or median (interquartile range (IQR)) for continuous variables. Group comparisons were made using Pearson’s chi-squared test for categorical variables and the Student’s *t*-test or Mann–Whitney *U*-test for continuous variables, as appropriate.

To evaluate the independent effect of prior statin use on outcome measures, we performed binary logistic regression analysis. Variables for adjustment in the multivariable analysis were selected if their *p*-values were <0.1 in comparisons whether they had prior statin use or not and if their associations with each outcome variable were clinically plausible. Crude and adjusted odds ratios (ORs) and 95% confidence intervals (CIs) were estimated.

Subgroup analysis was performed to evaluate the discrepancy of outcomes according to the intensity of statin. We described the crude rate of primary and secondary outcomes as an increase in statin intensity. In addition, to evaluate the heterogeneous effects of prior statin use according to stroke subtypes, we performed subgroup analyses (large artery atherosclerosis (LAA) versus cardioembolism (CE)).

As a sensitivity analysis, we performed propensity score matching (PSM) analysis when the imbalances of variables between the prior statin group and no prior statin group were significantly severe.

## 3. Results

Among 4779 patients with acute ischemic stroke who were hospitalized within 7 days of onset, we enrolled 385 patients receiving EVT in this study. The mean age was 70.2 ± 13.0 years, with men comprising 58.7%. Reperfusion therapy was EVT only in 50.6% and combined IVT and EVT in 49.4%. The overall successful reperfusion rate was 81.8% in this study.

Seventy-four patients (19.2%) were already prescribed statins before stroke onset in EVT-receiving patients. Of these, high-intensity statin was given to 16.2% (*n* = 12), and moderate-intensity statin was given to 83.8% (*n* = 62). No subjects were prescribed a low-intensity statin. In patients receiving a high-intensity statin, four received atorvastatin 80 mg, six received atorvastatin 40 mg, and two received rosuvastatin 20 mg. Prior statin users were likely to be older and tended to have more previous hypertension, hyperlipidemia, current smoking, atrial fibrillation, lower low-density lipoprotein level, and longer prothrombin time than the no prior statin group. In addition, the prior statin group used more previous antithrombotic agents. Stroke severity, acute stroke management, and time interval from arrival to puncture time were not different between the two groups (Table 1). With respect to reperfusion therapy, most comorbidities (prior stroke, hypertension, diabetes mellitus) were higher in the combined IVT and EVT group than in the EVT only group (Supplementary Table S2).

**Figure 1:**
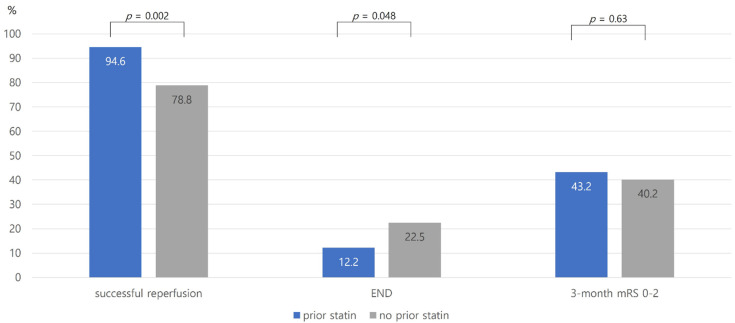
The proportion of outcomes according to prior statin use. Abbreviations: END, early neurologic deterioration; mRS, modified Rankin Scale.

**Figure 2:**
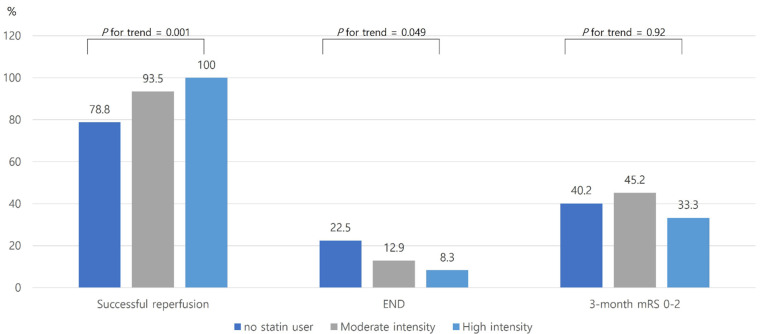
The proportion of outcomes according to the intensity of statins. Abbreviations: END, early neurologic deterioration; mRS, modified Rankin Scale.

**Figure 3:**
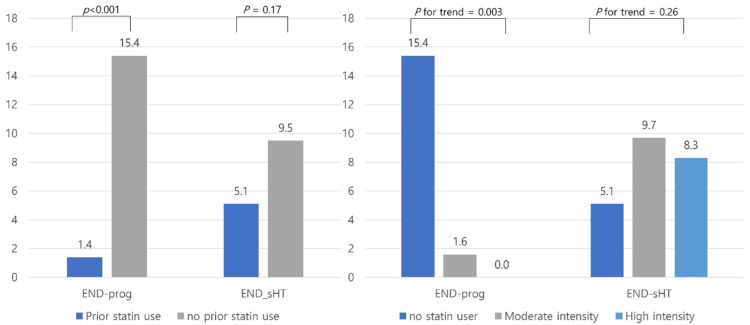
The proportion of occurrence of END according to prior statin use and the intensity of statins. Abbreviations: END-prog, Stroke progression; END-sHT. Symptomatic hemorrhagic transformation.

The successful reperfusion rate was higher in prior statin users than the no prior statin users (94.6% versus 78.8%, *p* = 0.002, Figure 1). Regarding the intensity of statins, subjects with high-intensity statins had more successful reperfusion in a dose–response manner (*p* for trend = 0.001, Figure 2). The occurrence of END was lower in prior statin users than no prior statin users (12.2% versus 22.5%, *p* = 0.048, Figure 1). Subjects receiving high-intensity statins had a lower occurrence of END in a dose–response manner (Figure 2). In addition, the proportion of END-prog was lower in prior statin users, especially in high-intensity statins. However, END-sHT was not different between the two groups (Figure 3). END-rec was not present in our database. A good functional outcome at 3 months was not different between the two groups (Figure 2). In addition, a good functional outcome was not different according to the intensity of statins.

In multivariate analysis, prior statin use increased the successful reperfusion rate after EVT (adjusted OR (95% CI) 5.31 (1.67–16.86), Table 2). Furthermore, prior statin use was significantly associated with a low occurrence of END and a good functional outcome at 3 months (Table 3, Supplementary Table S3). Regarding statin intensity, the OR of high-intensity statins for successful reperfusion was not calculated because all subjects receiving high-intensity statins had successful reperfusion. The associations between high-intensity statin and END or 3-month mRS 0 to 2 were attenuated compared with those of the moderate-intensity statin group.

Subgroup analysis according to stroke subtype showed dose–response relationships between statin intensity and successful reperfusion in patients with LAA only. Otherwise, in CE, successful reperfusion was not different regardless of prior statin use or statin intensity (Supplementary Figures S1–S3).

When the imbalances of variables between the two groups were severe, we performed PSM analysis to compare primary outcomes. PSM analysis showed that the successful reperfusion rate was still higher in the prior statin group than in the no prior statin group (Supplementary Table S4).

## 4. Discussion

The main findings of this multicenter study were as follows: (1) prior statin users, especially in the high-intensity statin group, had a higher successful reperfusion rate after EVT; (2) the occurrence of END was lower in the prior statin use group, especially in the high-intensity statin group; and (3) prior statin use could be a predictor for successful reperfusion, low occurrence of END, and good functional outcomes after EVT.

This study is the first to evaluate the impact of prior statin use on reperfusion state and stroke outcome after EVT. The overall rate of successful reperfusion in this study (81.8%) was comparable with previous EVT trials [2]. Approximately 95% of prior statin users experienced successful reperfusion after EVT in this study. Despite the small sample size, prior statin users with high-intensity statins had a 100% successful reperfusion rate. In a recent previous EVT study of prior antiplatelet use (*n* = 369), 84% had successful reperfusion after EVT [27]. Experimental studies showed that statin improved endothelial function via the upregulation of endothelial nitric oxide synthase, amelioration of glutamate-mediated excitotoxicity, and modulation of cytokine responses [28,29]. The result of subgroup analysis in LAA could robust this pathomechanism. In addition to its fibrinolytic effects, prior statin use before EVT might have had a positive effect on the successful reperfusion rate in our study. However, it is important to note that in our study, there were several comorbidities in the prior statin group. The controversial results of prior statin effect on stroke outcomes reported by previous studies of IVT treated patients can be explained by the high prevalence of comorbidities in the prior statin group. Nonetheless, our multivariate analysis showed that these vascular risk factors (except for age) did not affect reperfusion after EVT. Rather, we suggest that the burden of complications can be reduced when EVT is performed for patients with several comorbidities who are prescribed statins in real-world practice. Since the population of statin use consistently had increased according to the ACC/AHA cholesterol guidelines [30], our main findings could raise the interest of prior statin for predicting successful reperfusion.

Subgroup analysis showed that prior statin users with LAA had significant successful reperfusion after EVT, but not with CE. Statin therapy may be mainly attributable to plaque stabilization and the potential regression of atherosclerosis. These pleiotropic effects of statin seemed to be more beneficial in LAA patients receiving EVT. The previous studies showed that the reperfusion rate of LAA was lower than that of CE. Navigation of the guidewire or stent retriever through the atherosclerotic lesion and the tendency for re-occlusion due to in situ thrombosis hindered successful reperfusion in LAA after EVT [31]. Therefore, additional rescue therapy is frequently applied to LAA patients [32]. The results of our subgroup analysis showing different reperfusion rates according to stroke subtype support the feasibility of prior statin use in patients receiving EVT, especially in LAA. However, it should be noted that statins could be of benefit in cardioembolic stroke [33,34]. Previous studies of atrial fibrillation showed that statin therapy was associated with a better outcome, functional recovery, and long-term survival [35]. Additional pleiotropic effects (fibrinolytic, antithrombotic, and antioxidant effects) might have a positive impact on these outcomes in cardioembolic stroke patients. Therefore, we should be cautious when generalizing our positive results related to prior statin effects on the reperfusion state in LAA patients only.

Interestingly, prior statin users had a low occurrence of END, despite the high prevalence of several vascular risk factors in prior statin users. Notably, prior statin users had a lower occurrence of END-prog and tended to have a lower occurrence of END-sHT. These phenomena highlight the importance of the neuroprotective effects of statin in large artery occlusion patients. Otherwise, the prior statin users did not have a significantly good functional outcome at 3 months in our study, although prior statin increased good functional outcomes after EVT in multivariate analysis. This result was in accord with the previous largest study of IVT-treated patients addressing the negative prognostic value of prior statin use [17]. This might be explained by the attenuated neuroprotective effects of statins mediated via the neurotoxicity properties of tissue plasminogen activator, as well as the idea that several vascular risk factors might promote a negative outcome after IVT. Several comorbidities in the prior statin group might have affected the crude analysis in our study. In addition, other factors (functional status at discharge, discharge medications, and rehabilitation) might have affected the functional status at 3 months, and therefore, prior statin effects on the 3-month mRS might have been lost in the crude analysis. Nonetheless, our positive results in multivariate analysis on stroke outcomes in EVT patients suggest that prior statin use could be more beneficial, although prior statin users had several comorbidities. However, we presumed that unmeasured confounding might affect the delayed stroke outcome. Therefore, our results should be interpreted with caution because the effect of prior statin may be different depending on the phase of the stroke. Further studies are warranted to confirm our results.

High-intensity statin was expected to improve collateral flow and angiogenesis and restore endothelial damage [35,36]. However, because of concerns regarding the risk of intracranial hemorrhage by statins, especially high-intensity statins [37], patients with several comorbidities could be at high risk of hemorrhagic transformation after reperfusion therapy [38]. In our crude analysis, we showed a more successful reperfusion rate and lower END occurrence rate in patients with prior statin use with a dose–response relationship. Despite the lack of statistical significance, END-sHT tended to be lower in prior statin users, and the rate of END-sHT was attenuated in subjects with high-intensity statins. However, because of the insufficient sample size in the high-intensity statin group, the multivariate analysis did not demonstrate associations between statin intensity and outcomes. Nonetheless, regarding our crude results, we carefully suggest that high-intensity statins are not harmful, at least in patients receiving EVT. A large cohort study with high-intensity statins should be initiated.

Our results were based on a consecutive multicenter database, and there were several limitations. First, because this was a retrospective analysis, the individual duration of statin prescription prior to admission was not available. Second, our results could not show associations between high-intensity statins and outcomes in the multivariate analysis due to the small sample size. However, we showed better outcomes in high-intensity statin users in the crude analysis. Further study with a large sample size might reveal an association between high-intensity statin and outcomes. Third, our results cannot be generalized because we included subjects receiving combined IVT and EVT, which might impact the outcomes. However, the proportion of combined IVT and EVT was not different in the bivariate analysis. Fourth, collateral status was not included in the statistical analysis. According to the EVT protocol in three institutions, multiphasic CTA was performed to confirm collateral status, but there were 32 (8.3%) missing values for multiphasic CTA. Because there was relatively little missing data and the main aim of our study was to evaluate prior statin use on the successful reperfusion rate, we did not exclude subjects who lacked available collateral status data. Last, although we adjusted several variables to affect the outcomes, unmeasured confounding factors could hinder the generalization of our main findings. The outcomes after EVT could be influenced by procedure-related factors, including the type of stent retriever, workmanship, and procedure time, which were not available in our study.

## 5. Conclusions

Prior statin use before an ischemic stroke may increase successful reperfusion and good prognosis after EVT. Although we did not find significant associations between outcomes and intensity of statins, prior statin use may be beneficial and effective in the setting of EVT.

## Figures and Tables

**Table 1:** Baseline characteristics according to prior statin use and no prior statin use.

	No Prior Statin Use *n* = 311	Prior Statin Use *n* = 74	*p*-Value
Age, year (SD)	69.2 (13.6)	74.7 (9.4)	0.003 ^†^
Male, (%)	189 (60.8)	37 (50.0)	0.09 *
BMI, kg/cm^2^ (SD)	24.6 (19.9)	24.4 (3.8)	0.62 ^†^
Initial NIHSS, (IQR)	14 (9–18)	15 (11–18)	0.26 ^‡^
Stroke subtype, (%)			0.18 *
LAA	86 (27.7)	15 (20.3)	
CE	165 (53.1)	48 (64.9)	
others	60 (19.3)	11 (14.9)	
Interval from arrival to puncture time, min (IQR)	96.5 (73.0–137.0)	89.5 (72.0–113.0)	0.35 ^‡^
Interval from stoke onset to puncture time, min (IQR)	235.0 (146.0–400.0)	245.0 (137.0–385.0)	0.71 ^‡^
Prior stroke, (%)	49 (15.8)	28 (37.8)	<0.001 *
Hypertension, (%)	174 (55.9)	55 (74.3)	0.004 *
Diabetes mellitus, (%)	80 (25.7)	24 (32.4)	0.24 *
Hyperlipidemia, (%)	37 (11.9)	26 (35.1)	<0.001 *
Current smoking, (%)	47 (15.1)	3 (4.1)	0.01 *
Atrial fibrillation, (%)	159 (51.1)	48 (64.9)	0.03 *
Prior antithrombotics, (%)	75 (24.1)	57 (77.0)	<0.001 *
Antiplatelet, (%)	48 (15.4)	36 (48.6)	
Anticoagulation, (%)	30 (9.6)	25 (33.8)	
Reperfusion therapy, (%)			0.52 *
EVT only	160 (51.4)	35 (47.3)	
Combined IVT and EVT	151 (48.6)	39 (52.7)	
Antiplatelet in acute stage, (%)	174 (55.9)	43 (58.1)	0.74 *
Anticoagulationin acute stage, (%)	131 (42.1)	30 (40.5)	0.80 *
Statin in acute stage, (%)	237 (76.2)	57 (77.0)	0.88 *
White blood cell, uL/10^3^(SD)	8.92 (3.55)	8.47 (3.00)	0.04 ^†^
Hemoglobin, mg/dL (SD)	13.6 (2.0)	13.8(1.7)	0.29 ^†^
Creatinine, mg/dL (SD)	0.95 (0.61)	0.90 (0.29)	0.12 ^†^
Platelet count, uL/10^3^ (SD)	229.2 (85.2)	217.5 (72.5)	0.36^†^
LDL, mg/dL (SD)	100.4 (35.9)	87.2 (42.3)	0.02 ^†^
Glycated hemoglobin, (%)	6.2 (1.4)	6.3 (1.3)	0.74 ^†^
Prothrombin time, INR (SD)	1.06 (0.17)	1.16 (0.47)	<0.001 ^†^
CRP, mg/dL (SD)	9.1 (21.7)	11.3 (24.4)	0.08 ^†^
Initial random glucose, mg/dL (SD)	145.0 (58.3)	137.4 (52.7)	0.54 ^†^
Systolic blood pressure, mmHg (SD)	147.4 (26.1)	151.3 (25.7)	0.91 ^†^
Infarct volume, cm^3^ (IQR)	14.2 (3.5–50.2)	10.5 (1.9–44.2)	0.24 ^‡^

Abbreviation: SD, standard deviation; BMI, body mass index; NIHSS, National Institute Health of Stroke Scale; IQR, interquartile range; LAA, large artery atherosclerosis; CE, cardioembolism; EVT, endovascular treatment; IVT, intravenous thrombolysis; LDL, low-density lipoprotein; CRP, C-reactive protein. * Calculated using the chi-square test ^†^ Calculated using Student’s *t*-test ^‡^ Calculated using the Mann–Whitney *U* test.

**Table 2:** Multivariate analysis showing effect of prior statin use on successful reperfusion.

	Adjusted OR	95% CI	*p*-Value
Age	0.97	0.94–0.999	0.04
Male	1.65	0.88–3.10	0.12
Initial NIHSS	0.98	0.94–1.03	0.39
Stroke subtype			
Others	Reference		
CE	1.04	0.47–2.29	0.92
LAA	2.47	1.02–5.96	0.045
Prior stroke	1.07	0.49–2.34	0.86
HTN	0.83	0.44–1.57	0.56
Hyperlipidemia	2.05	0.79–5.36	0.14
Current smoking	0.88	0.36–2.17	0.78
Atrial fibrillation	1.06	0.47–2.42	0.88
Prior antithrombotics	0.98	0.48–2.01	0.97
White blood cell	0.99	0.91–1.08	0.85
Low density lipoprotein	0.97	0.99–1.004	0.33
Prothrombin time	0.52	0.18–1.49	0.52
C-reactive protein	1.001	0.99–1.01	0.94
Prior statin use	5.31	1.67–16.86	0.01
No user	Reference		
Moderate intensity statin	4.48	1.41–14.25	0.01
High intensity statin	-	-	-

Abbreviation: OR, odd ratio; CI, confidence interval; NIHSS, National Institute Health of Stroke Scale; LAA, large artery atherosclerosis; CE, cardioembolism; HTN, hypertension.

**Table 3:** Multivariate analysis showing effect of prior statin use on stroke outcomes.

	END		3-Month mRS 0–2	
	aOR	95% CI	aOR	95% CI
Prior stain use	0.38	0.17–0.89	2.03	1.06–3.91
Statin intensity				
No user	Reference		Reference	
Moderate	0.41	0.17–0.98	2.21	1.12–4.37
High	0.24	0.03–2.07	1.20	0.29–4.95

Abbreviation: END, early neurologic deterioration; mRS, modified Rankin Scale; aOR, adjusted odd ratio; CI, confidence interval.

## Data Availability

The data presented in this study are available on request from the corresponding author.

## Supplementary Materials

The following are available online at https://www.mdpi.com/article/10.3390/jcm10215147/s1, Figure S1: Successful reperfusion according to stroke subtypes, Figure S2: Successful reperfusion according to stroke subtypes and the intensity of statin, Figure S3: Study flowchart, Table S1: Statins according to intensity, Table S2: Baseline characteristics according to reperfusion therapy, Table S3: Multivariate analysis showing the effect of prior statin use on stroke outcomes, Table S4: Descriptive Statistics using Propensity Score Matching.

## References

[1] Powers W.J., Rabinstein A.A., Ackerson T., Adeoye O.M., Bambakidis N.C., Becker K., Biller J., Brown M., Demaerschalk B.M., Hoh B. (2018). 2018 Guidelines for the Early Management of Patients with Acute Ischemic Stroke: A Guideline for Healthcare Professionals from the American Heart Association/American Stroke Association. Stroke.

[2] Campbell B.C.V., Donnan G.A., Lees K.R., Hacke W., Khatri P., Hill M.D., Goyal M., Mitchell P.J., Saver J.L., Diener H.C. (2015). Endovascular stent thrombectomy: The new standard of care for large vessel ischaemic stroke. Lancet Neurol..

[3] Rubiera M., Alvarez-Sabin J., Ribo M., Montaner J., Santamarina E., Arenillas J.F., Huertas R., Delgado P., Purroy F., Molina C.A. (2005). Predictors of early arterial reocclusion after tissue plasminogen activator-induced recanalization in acute ischemic stroke. Stroke.

[4] Nie X., Pu Y., Zhang Z., Liu X., Duan W., Liu L. (2018). Futile Recanalization after Endovascular Therapy in Acute Ischemic Stroke. Biomed. Res. Int..

[5] Pan H., Lin C., Chen L., Qiao Y., Huang P., Liu B., Zhu Y., Su J., Liu J. (2021). Multiple-Factor Analyses of Futile Recanalization in Acute Ischemic Stroke Patients Treated with Mechanical Thrombectomy. Front. Neurol..

[6] Amarenco P., Labreuche J. (2009). Lipid management in the prevention of stroke: Review and updated meta-analysis of statins for stroke prevention. Lancet Neurol..

[7] Violi F., Calvieri C., Ferro D., Pignatelli P. (2013). Statins as antithrombotic drugs. Circulation.

[8] Tsunekawa T., Hayashi T., Kano H., Sumi D., Matsui-Hirai H., Thakur N.K., Egashira K., Iguchi A. (2001). Cerivastatin, a hydroxymethylglutaryl coenzyme a reductase inhibitor, improves endothelial function in elderly diabetic patients within 3 days. Circulation.

[9] Ovbiagele B., Saver J.L., Starkman S., Kim D., Ali L.K., Jahan R., Duckwiler G.R., Vinuela F., Pineda S., Liebeskind D.S. (2007). Statin enhancement of collateralization in acute stroke. Neurology.

[10] Wang S., Lee S.R., Guo S.Z., Kim W.J., Montaner J., Wang X., Lo E.H. (2006). Reduction of tissue plasminogen activator-induced matrix metalloproteinase-9 by simvastatin in astrocytes. Stroke.

[11] van der Most P.J., Dolga A.M., Nijholt I.M., Luiten P.G., Eisel U.L. (2009). Statins: Mechanisms of neuroprotection. Prog. Neurobiol..

[12] Laufs U., Gertz K., Huang P., Nickenig G., Bohm M., Dirnagl U., Endres M. (2000). Atorvastatin upregulates type III nitric oxide synthase in thrombocytes, decreases platelet activation, and protects from cerebral ischemia in normocholesterolemic mice. Stroke.

[13] Zhao H.D., Zhang Y.D. (2014). The effects of previous statin treatment on plasma matrix metalloproteinase-9 level in Chinese stroke patients undergoing thrombolysis. J. Stroke Cerebrovasc. Dis..

[14] Cappellari M., Bovi P., Moretto G., Zini A., Nencini P., Sessa M., Furlan M., Pezzini A., Orlandi G., Paciaroni M. (2013). The THRombolysis and STatins (THRaST) study. Neurology.

[15] Tsivgoulis G., Kadlecova P., Kobayashi A., Czlonkowska A., Brozman M., Svigelj V., Csiba L., Korv J., Demarin V., Vilionskis A. (2015). Safety of Statin Pretreatment in Intravenous Thrombolysis for Acute Ischemic Stroke. Stroke.

[16] Rocco A., Sykora M., Ringleb P., Diedler J. (2012). Impact of statin use and lipid profile on symptomatic intracerebral haemorrhage, outcome and mortality after intravenous thrombolysis in acute stroke. Cerebrovasc. Dis..

[17] Engelter S.T., Soinne L., Ringleb P., Sarikaya H., Bordet R., Berrouschot J., Odier C., Arnold M., Ford G.A., Pezzini A. (2011). IV thrombolysis and statins. Neurology.

[18] Hong K.S., Lee J.S. (2015). Statins in Acute Ischemic Stroke: A Systematic Review. J. Stroke.

[19] Kang J., Kim N., Park T.H., Bang O.Y., Lee J.S., Lee J., Han M.K., Park S.H., Gorelick P.B., Bae H.J. (2015). Early statin use in ischemic stroke patients treated with recanalization therapy: Retrospective observational study. BMC Neurol..

[20] Kim S., Choi H., Won C.W. (2020). Effects of Statin Use for Primary Prevention among Adults Aged 75 Years and Older in the National Health Insurance Service Senior Cohort (2002–2015). Ann. Geriatr. Med. Res..

[21] Son K.B., Bae S. (2019). Patterns of statin utilisation for new users and market dynamics in South Korea: A 13-year retrospective cohort study. BMJ. Open.

[22] Ko Y., Lee S., Chung J.W., Han M.K., Park J.M., Kang K., Park T.H., Park S.S., Cho Y.J., Hong K.S. (2014). MRI-based Algorithm for Acute Ischemic Stroke Subtype Classification. J. Stroke.

[23] Stone N.J., Robinson J.G., Lichtenstein A.H., Bairey Merz C.N., Blum C.B., Eckel R.H., Goldberg A.C., Gordon D., Levy D., Lloyd-Jones D.M. (2014). 2013 ACC/AHA guideline on the treatment of blood cholesterol to reduce atherosclerotic cardiovascular risk in adults: A report of the American College of Cardiology/American Heart Association Task Force on Practice Guidelines. J. Am. Coll. Cardiol..

[24] Dargazanli C., Fahed R., Blanc R., Gory B., Labreuche J., Duhamel A., Marnat G., Saleme S., Costalat V., Bracard S. (2018). Modified Thrombolysis in Cerebral Infarction 2C/Thrombolysis in Cerebral Infarction 3 Reperfusion Should Be the Aim of Mechanical Thrombectomy: Insights from the ASTER Trial (Contact Aspiration Versus Stent Retriever for Successful Revascularization). Stroke.

[25] Jeong H.G., Kim B.J., Yang M.H., Han M.K., Bae H.J. (2015). Neuroimaging markers for early neurologic deterioration in single small subcortical infarction. Stroke.

[26] Sims J.R., Gharai L.R., Schaefer P.W., Vangel M., Rosenthal E.S., Lev M.H., Schwamm L.H. (2009). ABC/2 for rapid clinical estimate of infarct, perfusion, and mismatch volumes. Neurology.

[27] Zhu F., Anadani M., Labreuche J., Spiotta A., Turjman F., Piotin M., Steglich-Arnholm H., Holtmannspotter M., Taschner C., Eiden S. (2020). Impact of Antiplatelet Therapy During Endovascular Therapy for Tandem Occlusions: A Collaborative Pooled Analysis. Stroke.

[28] Vaughan C.J., Delanty N. (1999). Neuroprotective properties of statins in cerebral ischemia and stroke. Stroke.

[29] Pezzini A., Del Zotto E., Volonghi I., Giossi A., Costa P., Padovani A. (2009). New insights into the pleiotropic effects of statins for stroke prevention. Mini Rev. Med. Chem..

[30] Kernan W.N., Ovbiagele B., Black H.R., Bravata D.M., Chimowitz M.I., Ezekowitz M.D., Fang M.C., Fisher M., Furie K.L., Heck D.V. (2014). Guidelines for the prevention of stroke in patients with stroke and transient ischemic attack: A guideline for healthcare professionals from the American Heart Association/American Stroke Association. Stroke.

[31] Sun B., Shi Z., Pu J., Yang S., Wang H., Yang D., Hao Y., Lin M., Ke W., Liu W. (2019). Effects of mechanical thrombectomy for acute stroke patients with etiology of large artery atherosclerosis. J. Neurol. Sci..

[32] Kang D.H., Kim Y.W., Hwang Y.H., Park S.P., Kim Y.S., Baik S.K. (2014). Instant reocclusion following mechanical thrombectomy of in situ thromboocclusion and the role of low-dose intra-arterial tirofiban. Cerebrovasc. Dis..

[33] Ntaios G., Papavasileiou V., Makaritsis K., Milionis H., Manios E., Michel P., Lip G.Y., Vemmos K. (2014). Statin treatment is associated with improved prognosis in patients with AF-related stroke. Int. J. Cardiol..

[34] Park H.K., Lee J.S., Hong K.S., Cho Y.J., Park J.M., Kang K., Lee S.J., Kim J.G., Cha J.K., Kim D.H. (2020). Statin therapy in acute cardioembolic stroke with no guidance-based indication. Neurology.

[35] Choi K.H., Seo W.K., Park M.S., Kim J.T., Chung J.W., Bang O.Y., Kim G.M., Song T.J., Kim B.J., Heo S.H. (2019). Effect of Statin Therapy on Outcomes of Patients with Acute Ischemic Stroke and Atrial Fibrillation. J. Am. Heart Assoc..

[36] Kureishi Y., Luo Z., Shiojima I., Bialik A., Fulton D., Lefer D.J., Sessa W.C., Walsh K. (2000). The HMG-CoA reductase inhibitor simvastatin activates the protein kinase Akt and promotes angiogenesis in normocholesterolemic animals. Nat. Med..

[37] Goldstein L.B., Amarenco P., Szarek M., Callahan A., Hennerici M., Sillesen H., Zivin J.A., Welch K.M., Investigators S. (2008). Hemorrhagic stroke in the Stroke Prevention by Aggressive Reduction in Cholesterol Levels study. Neurology.

[38] Saver J.L. (2007). Hemorrhage after thrombolytic therapy for stroke: The clinically relevant number needed to harm. Stroke.

