# Supplementary material for: Impact of Prior Statin Use on Reperfusion Rate and Stroke Outcomes in Patients Receiving Endovascular Treatment
Source: J Clin Med. 2021 Nov 2;10(21):5147. doi: 10.3390/jcm10215147 (PMC8584468; doi:10.3390/jcm10215147)

Supplementary materials

Table S1. Statins according to intensity.

	High intensity	Moderate intensity	Low intensity
Statins	Atorvastatin (40 mg) 80 mg<br>Rosuvastatin 20 mg (40 mg)	Atorvastatin 10 mg (20 mg)<br>Rosuvastatin (5 mg) 10 mg<br>Simvastatin 20–40 mg	Simvastatin 10 mg
		Pravastatin 40 mg (80 mg)<br>Lovastatin 40 mg (80 mg)<br>Fluvastatin XL 80 mg<br>Fluvastatin 40 mg BID<br>Pitavastatin 1–4 mg	Pravastatin 10–20 mg<br>Lovastatin 20 mg<br>Fluvastatin 20–40 mg

Table S2. Baseline characteristics according to reperfusion therapy.

	EVT only n=195	IVT and EVT n=190	p-value
Age, year (SD)	70.7 (13.2)	69.7 (12.9)	0.14†
Male, (%)	114 (58.5)	112 (58.9)	0.92*
BMI, kg/cm^2 (SD)	23.7 (3.8)	25.5 (25.2)	0.25†
Initial NIHSS, (IQR)	13 (8-18)	15 (11-18)	0.18‡
Stroke subtype, (%)			0.70*
LAA	49 (25.1)	52 (27.4)	
CE	112 (57.4)	101 (53.2)	
others	34 (17.4)	37 (19.5)	
Interval from arrival to puncture time, min (IQR)	95.5 (72-144)	91.5 (75-123)	0.34‡
Interval from stoke onset to puncture time, min (IQR)	375 (220-680)	170 (122-240)	<0.001‡
Prior stroke, (%)	48 (24.6)	29 (15.3)	0.03*
Hypertension, (%)	127 (65.1)	102 (53.7)	0.02*
Diabetes mellitus, (%)	63 (32.3)	41 (21.6)	0.02*
Hyperlipidemia, (%)	35 (17.9)	28 (14.7)	0.41*
Current smoking, (%)	27 (13.8)	23 (12.1)	0.65*
Atrial fibrillation, (%)	114 (58.5)	93 (48.9)	0.07*
Prior antithrombotics, (%)	75 (38.5)	57 (30.0)	0.09*
Prior statin (%)	35 (17.9)	39 (20.5)	0.61*
Successful reperfusion (%)	161 (82.6)	154 (81.1)	0.79*

3-month mRS 0 to 2 (%)	72 (36.9)	85 (44.7)	0.12*
END-prog (%)	32 (16.4)	17 (8.9)	0.03*
END-sHT (%)	0 (0.0)	23 (12.1)	<0.001*

* Calculated using the chi-square test

† Calculated using Student's t-test

‡ Calculated using the Mann–Whitney U test

Table S3. Multivariate analysis showing the effect of prior statin use on stroke outcomes.

	END			3-month mRS 0 to 2		
	OR	95% CI	p	OR	95% CI	p
Age	1.02	0.99-1.04	0.24	0.97	0.95-0.99	0.01
Male	0.81	0.46-1.43	0.46	1.38	0.83-2.29	0.21
Initial NIHSS	1.001	0.96-1.05	0.95	0.92	0.88-0.96	<0.001
Stroke subtype						
Others		reference			reference	
CE	1.14	0.53-2.47	0.74	0.95	0.47-1.92	0.88
LAA	0.48	0.21-1.13	0.09	2.23	1.03-4.86	0.04
Prior stroke	0.87	0.43-1.76	0.7	0.79	0.43-1.48	0.47
HTN	1.38	0.75-2.55	0.3	0.61	0.37-1.02	0.06
Hyperlipidemia	0.85	0.40-1.82	0.68	1.07	0.57-1.98	0.84
Current smoking	0.77	0.31-1.89	0.57	1.42	0.70-2.90	0.33
Atrial fibrillation	1.19	0.54-2.60	0.67	0.72	0.36-1.43	0.34
Prior antithrombotics	1.24	0.65-2.39	0.52	0.9	0.50-1.61	0.71
WBC	0.96	0.88-1.04	0.27	1.04	0.97-1.10	0.30

LDL	0.997	0.99-1.004	0.36	1.00	0.99-1.004	0.18
PT	0.67	0.17-2.67	0.57	0.36	0.11-1.19	0.10
CRP	1.003	0.99-1.02	0.54	0.99	0.98-1.004	0.18
Prior stain use	0.38	0.17-0.89	0.03	2.03	1.06-3.91	0.03
No user		reference			reference	
Moderate-intensity statin	0.41	0.17-0.98	0.046	2.21	1.12-4.37	0.02
High-intensity statin	0.24	0.03-2.07	0.19	1.20	0.29-4.95	0.80

Table S4. Descriptive Statistics using Propensity Score Matching.

	No prior statin use<br>n=74	prior statin use<br>n=74	p-value
Age, year (SD)	74.4 (10.5)	74.7 (9.4)	0.86
Male, (%)	37 (50.0)	37 (50.0)	0.09
BMI, kg/cm^2 (SD)	23.4 (3.8)	24.4 (3.8)	0.12
Initial NIHSS, (IQR)	12.9 (5.7)	14.5 (5.7)	0.08
Stroke subtype, (%)			0.70
LAA	43 (58.1)	48 (64.9)	
CE	13 (17.6)	11 (14.9)	
others	18 (24.3)	15 (20.3)	
Prior stroke, (%)	27 (36.5)	28 (37.8)	1.00
Hypertension, (%)	56 (75.7)	55 (74.3)	1.00
Diabetes mellitus, (%)	26 (35.1)	24 (32.4)	0.86
Hyperlipidemia, (%)	14 (18.9)	26 (35.1)	0.04
Current smoking, (%)	2 (2.7)	3 (4.1)	1.00
Atrial fibrillation, (%)	42 (56.8)	48 (64.9)	0.40

Prior antithrombotics, (%)	55 (50.0)	36 (48.6)	0.85
Prior antiplatelets (%)	37 (50.0)	36 (48.6)	1.00
Prior anticoagulants (%)	21 (28.4)	25 (33.8)	0.59
White blood cell, uL/10^3(SD)	9.0 (3.8)	8.5 (3.0)	0.37
Hemoglobin, mg/dL (SD)	13.1 (2.0)	13.8 (1.7)	0.03
Creatinine, mg/dL (SD)	1.0 (0.7)	0.9 (0.3)	0.23
Platelet count, uL/10^3 (SD)	217.6 (66.3)	217.5 (72.5)	0.36[†]
LDL, mg/dL (SD)	92.7 (34.3)	87.2 (42.3)	0.38
Glycated hemoglobin, (%)	6.2 (1.3)	6.3 (1.3)	0.56
Prothrombin time, INR (SD)	1.1 (0.3)	1.2 (0.5)	0.46
CRP, mg/dL (SD)	10.7 (18.9)	11.3 (24.4)	0.88
Initial random glucose, mg/dL (SD)	144.0 (59.8)	136.0 (46.1)	0.37
Systolic blood pressure, mmHg (SD)	146.9 (20.0)	151.3 (25.7)	0.25
Infarct volume, cm^3	43.2 (6.6)	64.0 (12.4)	0.22
Outcomes			
Successful reperfusion	58 (78.4)	70 (94.6)	0.01
END-prog	16 (21.6)	1 (1.4)	<0.001
3-month mRS 0-2	29 (39.2)	32 (43.2)	0.74

Figure S1. Successful reperfusion according to stroke subtypes.

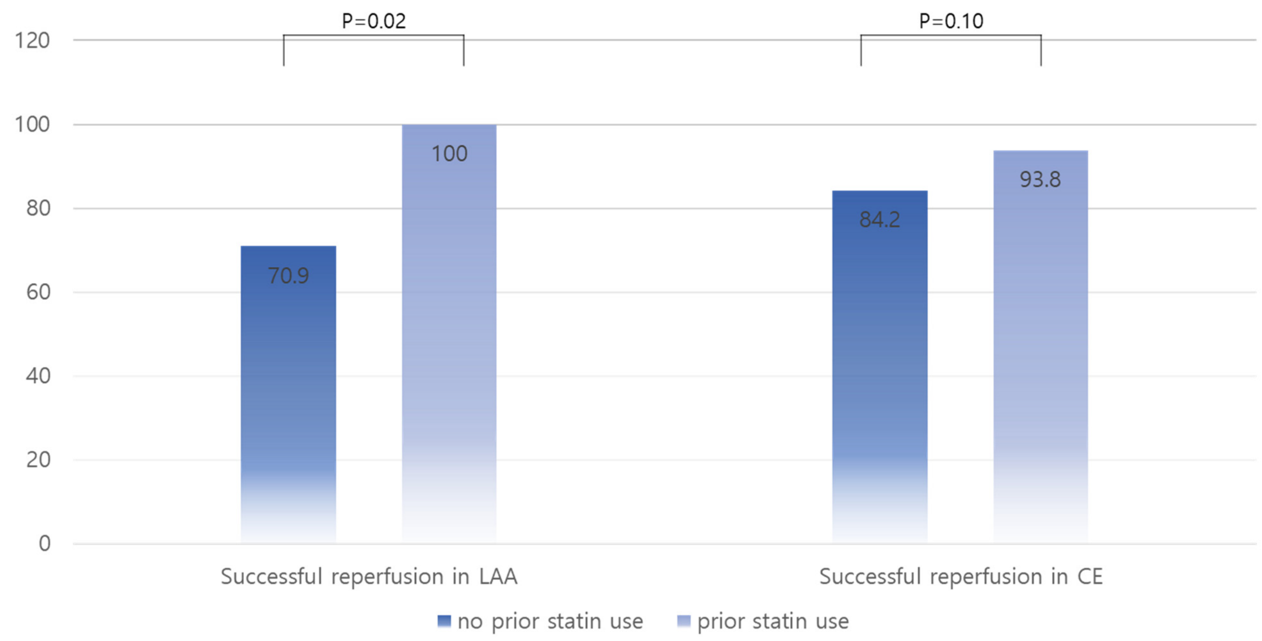

Figure S2. Successful reperfusion according to stroke subtypes and the intensity of statin.

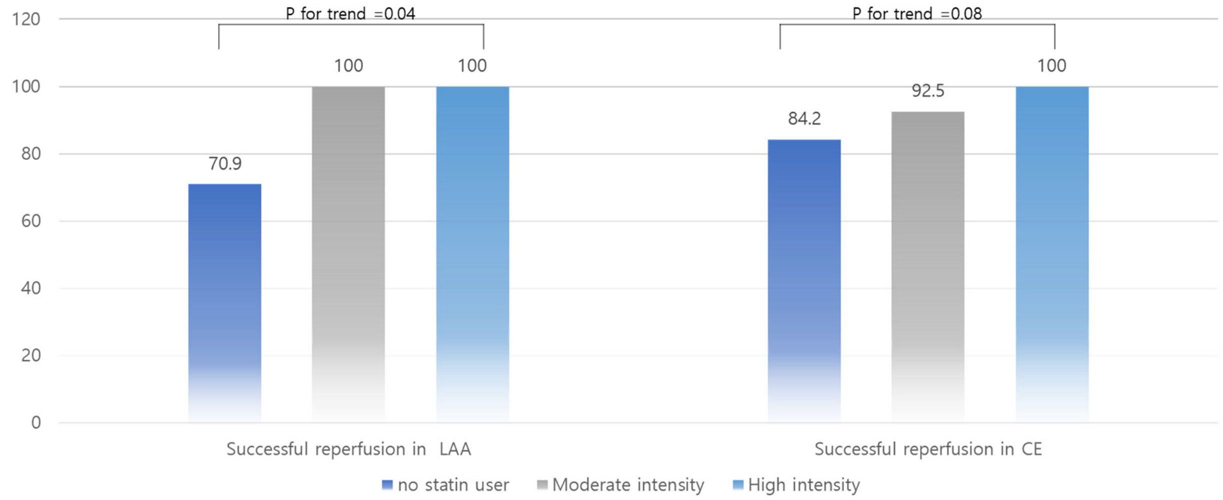

Figure S3. Study flowchart.

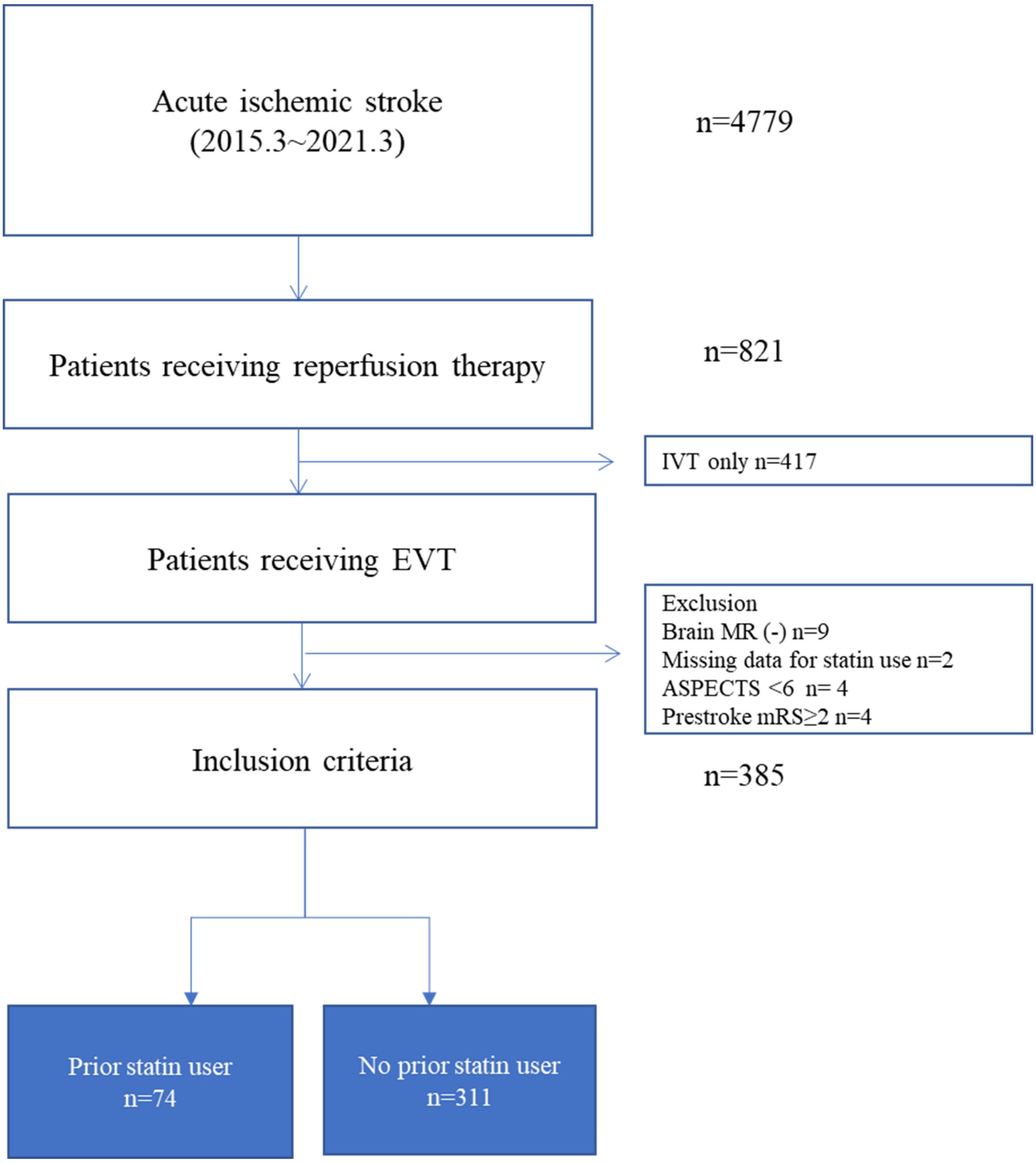

Supplement: Supplementary file 1 [file jcm-10-05147-s001.zip › jcm-1413690-supplementary.pdf]
